# An Unusual Initial Presentation of Gout at the Wrist: Extensor Tenosynovitis With Dual-Energy CT and MRI Correlation

**DOI:** 10.7759/cureus.94385

**Published:** 2025-10-12

**Authors:** Kwan Kit Chan, Wai Hoi Chan

**Affiliations:** 1 Department of Radiology, North District Hospital, Hong Kong, HKG; 2 Department of Orthopaedics and Traumatology, Princess Margaret Hospital, Hong Kong, HKG

**Keywords:** dual-energy ct, gout, mri, tenosynovitis, wrist

## Abstract

Gout involving the wrist as an initial presentation is uncommon, especially when manifesting as extensor tenosynovitis with tophaceous deposits. We present the case of a 34-year-old male with chronic wrist pain and a dorsal wrist mass whose diagnosis was established through multimodal imaging, including dual-energy CT (DECT) and MRI. DECT effectively identified monosodium urate (MSU) crystal deposits in the extensor tendons, while MRI demonstrated tenosynovitis and tophus infiltration, guiding surgical synovectomy. Postoperative recovery was favourable with the resolution of symptoms. This report highlights the diagnostic challenges of wrist gout and underscores the value of advanced imaging techniques in early detection and operative planning.

## Introduction

Gout is a common inflammatory arthropathy affecting approximately 3.9% of adults, characterized by the deposition of monosodium urate (MSU) crystals in joints and soft tissues [1]. In the upper extremity, tophi are most often found in the subcutaneous tissues, particularly around the elbow and the proximal interphalangeal joints [2]. Wrist gout as an initial presentation is relatively uncommon and accounts for only about 0.8-2% of all gout cases [3].

Wrist gout as an initial presentation is diagnostically challenging due to its rarity, atypical presentation, and overlap with other joint diseases and arthritis that can cause wrist pain. Traditional imaging modalities may not adequately demonstrate MSU crystal deposits, making dual-energy CT (DECT) an invaluable diagnostic tool [4]. MRI is also helpful in the diagnosis of gout at uncommon sites or early in the disease, before bone damage appears [5]. We present a case of chronic extensor tenosynovitis secondary to tophaceous gout in a young adult male, emphasizing the diagnostic value of multimodal imaging, including DECT and MRI, in establishing the diagnosis and guiding surgical management.

## Case presentation

A 34-year-old right-handed male patient presented with a four-year history of chronic right wrist pain and a two-year history of palpable mass on the dorsal aspect of his right wrist. He was a smoker and an occasional alcohol consumer. The pain was episodic in nature with functional limitations characterized by restricted wrist extension when fingers were extended, though he could extend his wrist normally when fingers were flexed. No other joint pain or mass at joints was reported by the patient. His father had gout and hyperuricemia.

Physical examination revealed a mobile mass on the dorsal wrist that moved distally with finger flexion and proximally to the level of the distal margin of the extensor retinaculum with finger extension. There was no focal tenderness or signs of acute inflammation. Finger extension was full without finger drop. Laboratory investigations showed elevated serum uric acid at 0.72 mmol/L (normal range: 0.25-0.52 mmol/L). C-reactive protein, white blood cell count, rheumatoid factor, and cyclic citrullinated peptide antibodies were normal. Creatinine was mildly elevated at 116 umol/L (normal range: 64-104 umol/L) (Table 1).

**Table 1:** Blood test results of the patient

Blood test	Result	Normal range
Uric acid	0.72 mmol/L	0.25-0.52 mmol/L
White blood cell count	7.4 x10^9^/L	4.0-9.7 x10^9^/L
C-reactive protein	3.1 mg/L	<5.0 mg/L
Creatinine	116 umol/L	64-104 umol/L
Rheumatoid factor	<15.9 IU/mL	<15.9 IU/mL
Cyclic citrullinated peptide antibodies	<1 RU/mL	<5 RU/mL

**Figure 1:**
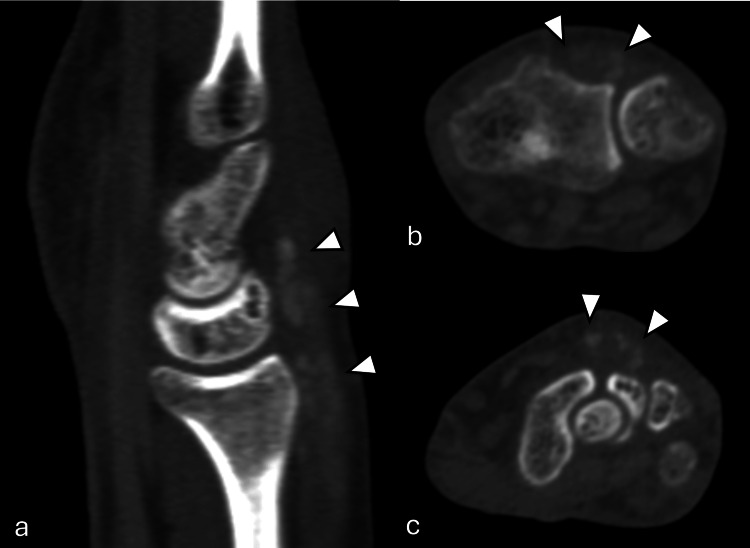
CT right wrist in bone window CT right wrist in bone window with sagittal (a) and axial (b, c) planes. Soft tissue swelling with high-density foci (arrowheads) over the dorsal aspect of the wrist, involving the extensor tendons, corresponds to intratendinous gouty tophi CT: computed tomography

**Figure 2:**
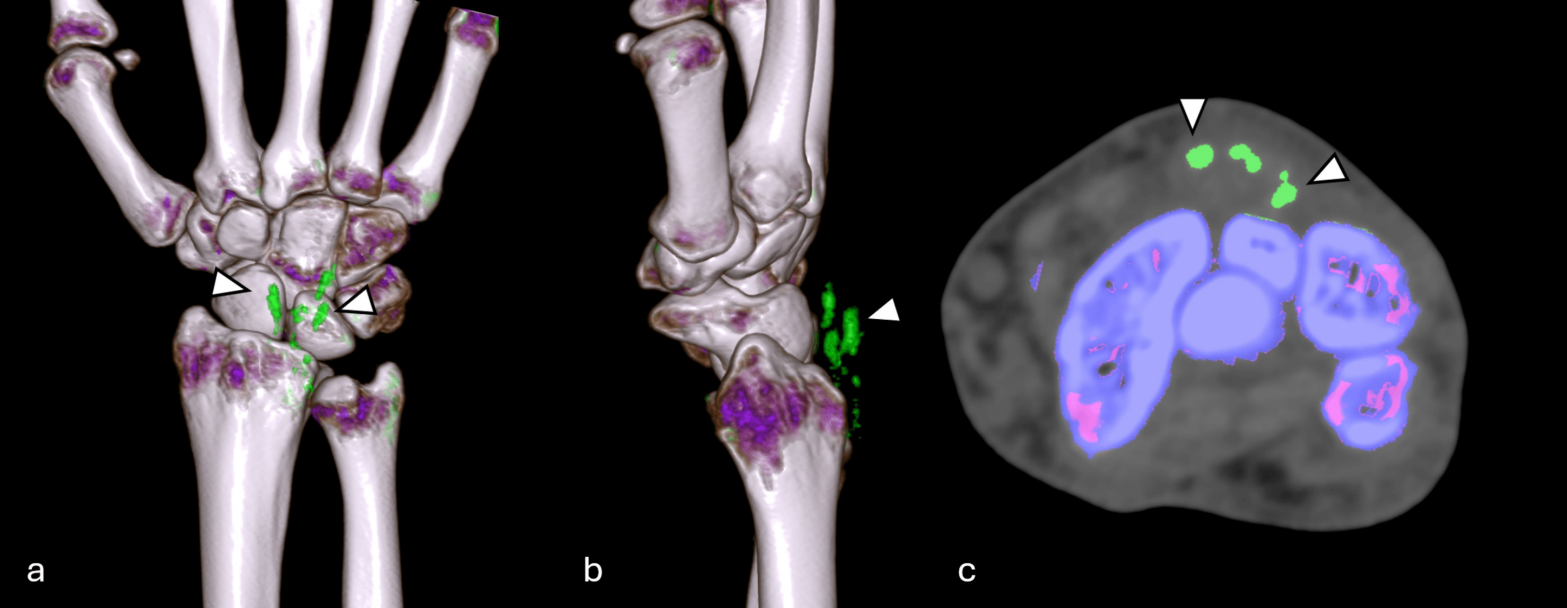
Dual-energy CT of the right wrist Dual-energy CT of the right wrist with 3D reconstruction (a, b) and at the axial plane (c). Dual-energy CT with color coding demonstrated the presence of uric acid deposits at the fourth extensor compartment of the wrist, consistent with gouty tophi (arrowheads) CT: computed tomography

DECT examination (uric acid/calcium pair; Hounsfield unit (HU) threshold: 150; iodine ratio: 1.36) revealed soft tissue swelling with high-density foci over the dorsal aspect of the right wrist, involving the extensor tendons (Figure 1). Color-coded dual-energy analysis demonstrated the presence of uric acid deposits consistent with gouty tophi (Figure 2). No similar findings were identified in the carpal tunnel or the rest of the wrist joint.

**Figure 3:**
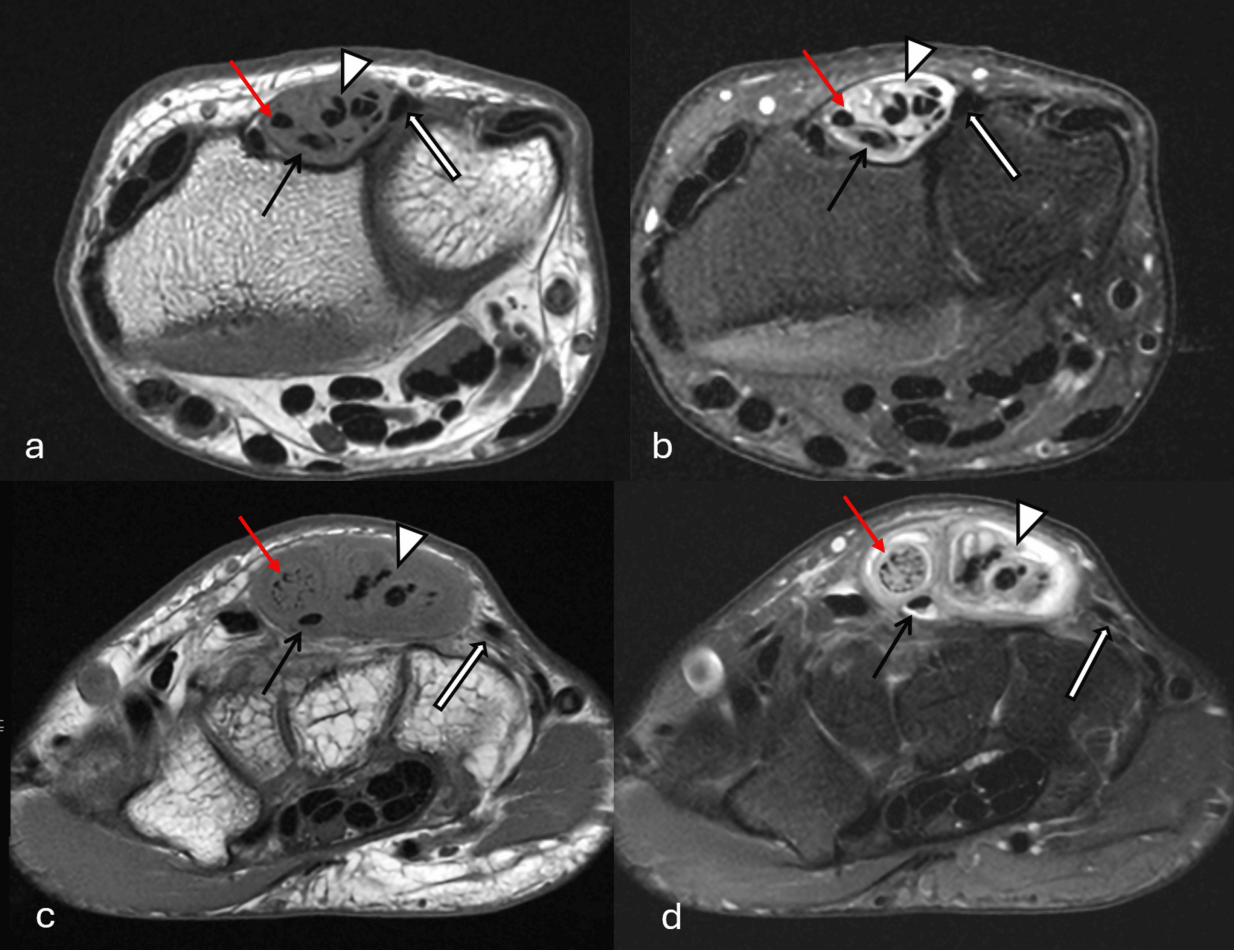
T1W and T2W fat-suppressed images of MRI wrist T1-weighted (T1W) axial images (a, c) and T2-weighted (T2W) fat-suppressed images (b, d) of the MRI right wrist of the patient. They demonstrated grossly thickened extensor digitorum tendons with moderate-volume sheath fluid and sheath thickening. Heterogeneous para- and intratendinous signal changes were observed, compatible with tenosynovitis and gouty tophus infiltration. Note the significant tendon thickening at the extensor digitorum communis tendons to the second digit (red arrows), thickened extensor digitorum communis tendons to the third, fourth, and fifth digits with suspect longitudinal tears (arrowheads). Preserved extensor indicis tendon (black arrows) and extensor digiti minimi tendon (block arrows). MRI provided a detailed visualization of the anatomy. The MRI results were consistent with the intraoperative findings MRI: magnetic resonance imaging

**Figure 4:**
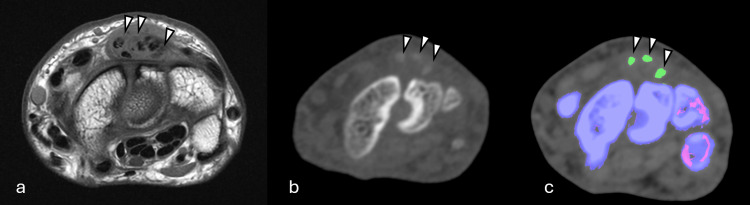
MRI, CT and Dual-energy CT images at the same level of the wrist Gouty tophi (arrowheads) appeared as low to intermediate signal in T1-weighted (T1W) MRI (a) and as mildly hyperdense foci in CT (b). They were easily picked up in dual-energy CT (c) MRI: magnetic resonance imaging; CT: computed tomography

MRI of the right wrist demonstrated grossly thickened extensor digitorum tendons with moderate-volume sheath fluid and sheath thickening. Heterogeneous para-tendinous and intratendinous signal changes were observed, compatible with tenosynovitis and gouty tophus infiltration (Figure 3), corroborating the DECT findings (Figure 4).

Based on the clinical presentation, elevated uric acid, family history, together with the DECT and MRI findings, tophaceous gout affecting the wrist extensor tendons was suspected. Allopurinol was started, and a low-purine diet was recommended. Despite medical management with allopurinol, which successfully reduced uric acid levels to 0.57 mmol/L, the patient continued to experience a similar frequency of joint pain attacks and persistent limitation in wrist extension. Consequently, surgery was offered.

Intraoperative exploration revealed copious synovitis predominantly involving the fourth extensor compartment with visible gouty tophaceous material. The extensor digitorum communis tendons to the second, third, fourth, and fifth digits (EDC2, EDC3, EDC4, EDC5) showed approximately 30% partial tears with unhealthy-appearing tendons that were grossly thickened and irregular in appearance, particularly in the area just distal to the extensor retinaculum. The extensor indicis (El) and extensor digiti minimi (EDM) are intact. No signs of infection were present. Debridement of the gouty tophaceous materials with surgical synovectomy of the affected synovium was performed.

**Figure 5:**
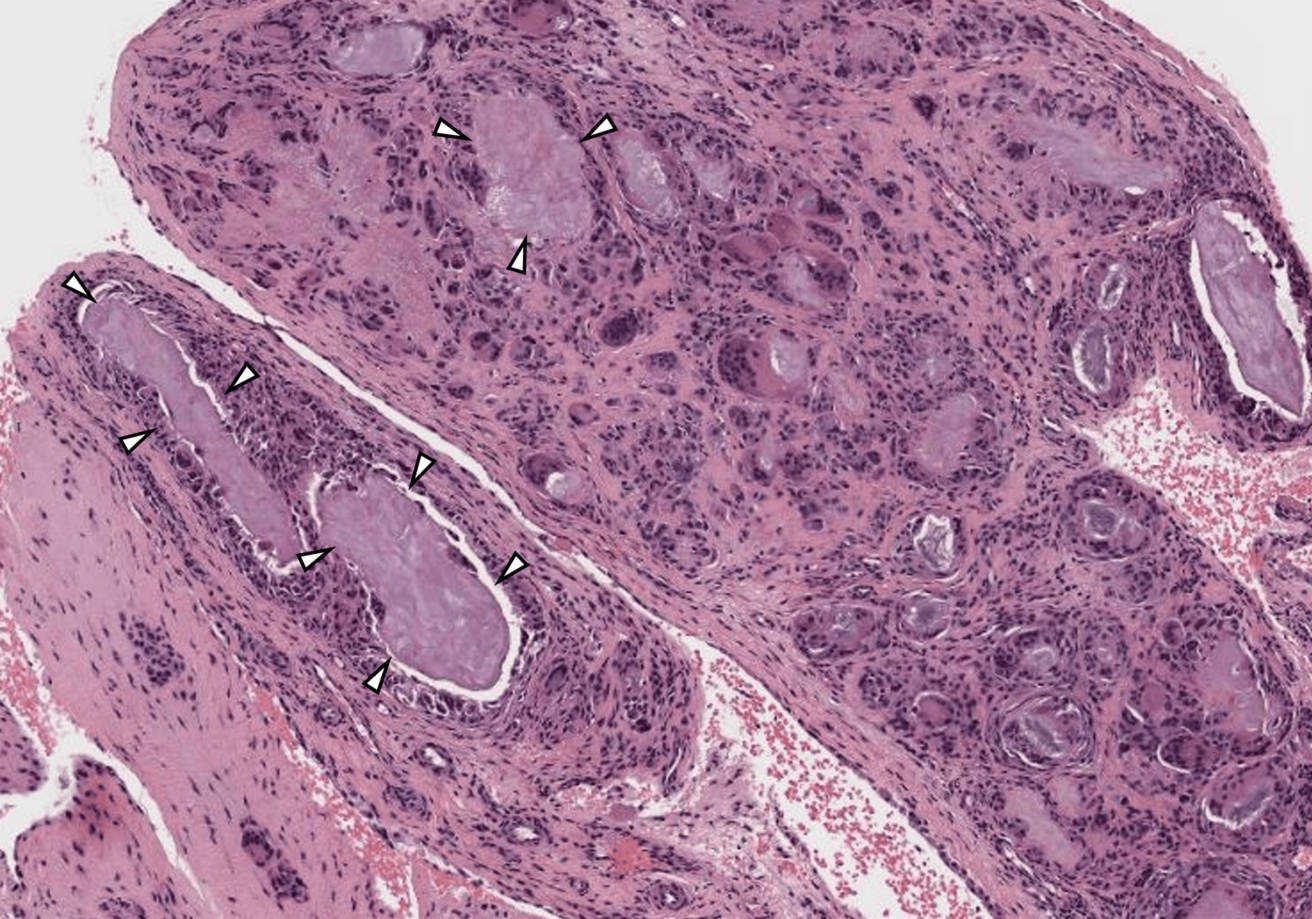
Histopathological examination of the resected synovial tissue Histopathological examination (H&E staining) of the resected synovial tissue revealed deposits of acellular pale-staining fibrillary material (arrowheads), admixed with multinucleated giant cells, compatible with gout

Histopathological examination of the resected synovial tissue revealed deposits of acellular pale-staining fibrillary material admixed with multinucleated giant cells with overall features compatible with gout (Figure 5).

Following rehabilitation, the patient regained a full range of motion in the affected wrist. Postoperative follow-up demonstrated resolution of the mechanical symptoms and improved functional capacity.

## Discussion

Gout affecting the wrist is uncommon, with wrist tendon urate deposition found in 5.4% of gouty patients in a study involving 148 patients [6]. The fourth and fifth extensor compartments are among the more commonly affected sites in the hand/wrist, with reported involvement rates of 55.1% and 53.8% respectively, of non-acute hand/wrist gout patients [7]. Traditional radiographic imaging has limited utility in early gout diagnosis, typically only revealing changes such as bone erosions, joint destruction, and tophi after 7-10 years of disease progression [6]. Recent advanced imaging modalities, including DECT and MRI, have enhanced both early detection capabilities and diagnostic problem-solving in gout evaluation.

DECT proved invaluable in this case by definitively identifying MSU crystal deposits within the wrist extensor tendons. This advanced imaging technique utilizes the material-specific attenuation differences of urate crystals at different energy levels (usually at 80 kV and 140 kV), allowing accurate detection and color-coding of MSU deposits [7]. DECT is a highly sensitive and specific non-invasive method for detecting MSU crystals, with reported sensitivity of 88% and specificity of 90% for gout diagnosis [8]. The high sensitivity and specificity of DECT make it particularly useful in cases where joint aspiration is not feasible for definitive diagnosis or when crystal deposition occurs in extra-articular locations such as tendons and ligaments [9].

On MRI, tophi usually appear as low signal on T1-weighted images and variable intermediate to high signal on T2-weighted images, reflecting cellular tissue around the crystals. The vascularity of this tissue affects the extent of contrast enhancement, while calcifications within the tophus cause low T2 signal areas [5]. MRI also provided detailed visualization of the associated soft tissue changes, including tendon thickening, synovial proliferation, and the extent of tenosynovitis [10]. This multimodal imaging approach enabled comprehensive preoperative planning and informed the surgical decision-making process. In this case, the surgical findings correlated well with the imaging studies, demonstrating the efficacy of DECT and MRI in assisting preoperative planning.

Surgical indications for gout in the hand and wrist include severe tenosynovitis, tendon infiltration by tophi, and significant loss of joint or tendon function, as observed in our patient. Surgery involves the excision of inflamed synovium and removal of tophaceous deposits to restore function and reduce symptoms, particularly helpful when medical management fails [11]. Nonsteroidal anti-inflammatory drugs (NSAIDs), colchicine, and corticosteroids (oral or injected) are the primary treatments to reduce pain and swelling during acute gout attacks. For long-term management of chronic gout, urate-lowering drugs like allopurinol or uricosurics reduce tophi and prevent flare-ups [12]. In severe or resistant cases, biologics and targeting agents such as anti-interleukin-1 may be used [13].

## Conclusions

Wrist involvement as the initial presentation of gout poses a diagnostic challenge due to its rarity and nonspecific clinical signs. Traditional imaging is often inadequate in early disease stages. DECT combined with MRI offers complementary advantages in detecting urate crystals and associated soft tissue changes. Awareness of this uncommon presentation and the role of advanced imaging can aid in the timely diagnosis and improved patient outcomes.
